# A protocol for wide-scope non-target analysis of contaminants in small amounts of biota using bead beating tissuelyser extraction and LC-HRMS

**DOI:** 10.1016/j.mex.2020.101193

**Published:** 2021-01-05

**Authors:** R. Gil-Solsona, S. Rodriguez-Mozaz, M.S. Diaz-Cruz, A. Sunyer-Caldú, T. Luarte, J. Höfer, C. Galbán-Malagón, P. Gago-Ferrero

**Affiliations:** a Catalan Institute for Water Research, Girona, Spain; b Institute of Environmental Assessment and Water Research (IDAEA) Severo Ochoa Excellence Center, Spanish Council for Scientific Research (CSIC), Jordi Girona 18-26, E-08034 Barcelona, Spain; c University of Girona, Girona, Spain; d Doctorado en Medicina de la Conservación, Facultad de Ciencias de la Vida, Universidad Andres Bello, Chile; e Anillo de Investigación en Ciencia Antártica, POLARIX, Santiago de Chile, Chile; f Escuela de Ciencias del Mar, Facultad de Ciencias del Mar y Geografía, Pontificia Universidad Católica de Valparaíso, Chile; g Centro FONDAP de Investigación Dinámica de Ecosistemas Marinos de Altas Latitudes (IDEAL), Valdivia, Chile; h GEMA Center for Genomics, Ecology and Environment, Universidad Mayor, Huechuraba, Chile

**Keywords:** Biota, Non-target screening, Suspect screening, Bead beating, Mixed-mode solid phase extraction, HRMS

## Abstract

This work describes a robust and powerful method for wide-scope target and non-target analysis of xenobiotics in biota samples based on bead beating tissuelyser extraction, solid phase extraction (SPE) clean-up and further detection by liquid chromatography coupled to high resolution mass spectrometry (LC-HRMS). Unlike target methodologies, non-target methods usually aim at determining a wide range of still unknown substances with different physicochemical properties. Therefore, losses during the extraction process were minimised. Apart from that, the reduction of possible interferences showed to be necessary to expand the number of compounds that can be detected. This was achieved with an additional SPE clean-up step carried out with mixed-bed multi-layered cartridges. The method was validated with a set of 27 compounds covering a wide range of physicochemical properties, and further applied to the analysis of krill and fish samples.

• The bead beating extraction was efficient for a wide range of organic pollutants in small quantities of biota samples.
• Multi-layered solid phase extraction clean-up yield a wide xenobiotics coverage reducing matrix effects.
• Method validation with 27 compounds led to a suitable method for non-target analysis of organic pollutants in biota.

## Graphical abstract

**Figure:**
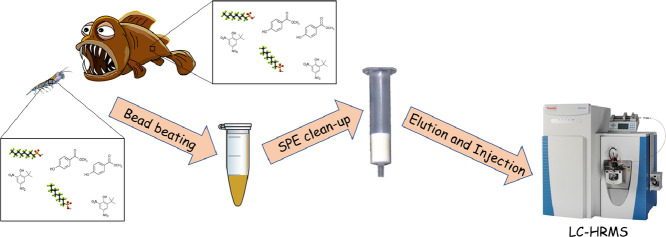

**Specifications Table**

**Table:** 

Subject Area:	Analytical chemistry, Molecular Biology and Environmental sciences
More specific subject area:	Organic contaminants
Method name:	Non-target wide-scope analysis of organic contaminants for small quantities of biota samples
Name and reference of original method	Impact of fullerenes in the bioaccumulation and biotransformation of venlafaxine, diuron and triclosan in river biofilms [1]; Extended Suspect and Non-Target Strategies to Characterize Emerging Polar Organic Contaminants in Raw Wastewater with LC-HRMS/MS [2]

## Method details

### Background

The development of non-target methodologies for the determination of organic contaminants that are not covered by existing target methodologies in complex biological samples is an urgent need. Target methods can only cover a small proportion of the compounds that can cause unwanted effects in the environment. Therefore, non-target strategies, where no particular chemicals are being searched for, are necessary to obtain a broader picture and identify new potentially hazardous compounds. In this sense, liquid chromatography coupled to high resolution mass spectrometry (LC-HRMS) has dramatically increased the opportunities for the detection of polar organic contaminants in complex samples [3]. However, the development of protocols for the extraction of many compounds covering a large range of physicochemical properties in biological matrices (e.g. biota) is still challenging. Advances in both extraction and clean-up protocols (to reduce interferences) for non-target analysis are needed.

In this work, an extraction method based on bead beating of small biota samples [1] was combined with a non-discriminant clean-up strategy for the non-target analysis of a wide-scope of organic pollutants [2]. In order to test its effectiveness for a wide range of organic contaminants the method was validated with a set of 27 compounds (logP comprised between −1.16 and 6.97) including pharmaceuticals, personal care products, herbicides, food additives and other industrial chemicals.

*Chemicals, reagents and other materials*

*Material for bead beating process*

Freeze-dryer (Lyo-alpha 6–80 Telstar)
Ceramic mortar
Milli-Q water (Millipore)
Ethanol for cleaning (Schalab)
Precision balance (Mettler Toledo)
Tissuelyzer Virtex-Genie 2 (MoBio Laboratorios, INC)
Centrifuge 5418R (Eppendorf)
2 mL extraction tubes (Deltalab)
Zirconium beads (Precellys)
Acetonitrile (HPLC-grade, Fischer Scientific)
Citric acid anydrous (Scharlab)
Tri-Sodium citrate 2-hydrate (Sharlab)
Water (HPLC-grade, Fischer Scientific)
Glass tubes (10 mL)
Glass bottles (150 mL)

*Material for Solid Phase Extraction clean-up protocol*

Empty SPE tubes 6cc, Polypropylene (Phenomenex)
Frits for 6cc SPE tubes, 20 µm (Phenomenex)
Sepra ZT (30 µm, 85 Å) powder (Phenomenex)
Sepra ZTL-WCX (100 µm, 300 Å) powder (Phenomenex)
Sepra ZTL-WAX (115 µm, 330 Å) powder (Phenomenex)
Isolute ENV+ powder (Biotage)
Vacuum collector HyperSep (Thermo Scientific)
Ethyl acetate 99.6% (Acros Organics)
Methanol (HPLC-grade, Merck)
Ammonia solution 32% (Merck)
Formic acid 88–90% (Merck)
Vacuum pump

*Material for dryness and reconstitution of the extracts*

ReactiVap (Thermo Scientific)
Nitrogen >99.9% (Linde Gas)
HPLC vials (Waters)
Micropipette 100–1000 µL (Thermo-Scientific)
Vortex 3 agitator (IKA Vortex)
Methanol (HPLC-grade, Merck)
Water (HPLC-grade, Merck)

### Sampling and sample pre-treatment

All samples were collected during the Austral Summer International Krill Synoptic Survey on board the RV Kronprins-Haakon and the RV Cabo de Hornos [4], [5], [6]. Krill and fish samples were collected in the Bransfield Strait (Antarctic Peninsula) and the South Scotia Sea,using a 42 m long macroplankton trawl, with a 36 m^2^ mouth opening, and a 3 mm mesh light [4,5]. From each catch a subsample of 20–30 krill and 2–3 lantern-fish individuals were collected randomly and wrapped in aluminum foil envelopes before freezing them at −20 °C.

Frozen samples were later transported to laboratory and freeze-dried overnight. Once the constant weight was reached, they were taken with stainless steel tweezers and placed in aluminum foil envelopes until the extraction and chemical analysis.

### Analytical protocol

The extraction protocol was adapted from Santos et al. [1]. For both krill and fish, 0.1 g of dried matrix was used.

1. Weight 1 g of zirconium beads and add them to the extraction tube.
2. Homogenize the samples in a ceramic mortar (previously cleaned with Milli-Q water, ethanol and dried).
3. Weight 0.1 g of each sample and add them into the extraction tube.
4. Add surrogate standards solution (see **Note #1)**. Allow evaporation of solvent 30–60 min.
5. Add 1 mL of the extraction mixture composed by citrate buffer and acetonitrile (see **Note #2**).
6. Carry out the sample extraction using tissuelyser (total time: 30 s, power: 5.5). Cryolys can be used instead of Precellys to avoid sample heating during the extraction.
7. Centrifuge the samples (11.000 *rcf* for 10 min).
8. Transfer the supernatant into a glass tube.
9. Repeat steps 4–7 two times and collect the supernatant always in the same tube.
10. Reduce the volume of the extracts (to 50%, approx. 1.5 mL) using a N_2_ evaporator in order to eliminate the excess of organic solvent.
11. Place the extract in a glass bottle with 100 mL of HPLC-grade water at pH 6.5 adjusted with ammonia and formic acid.
12. Clean the glass tubes with 3 mL of HPLC-grade water (three times).
13. Stabilize the mixed-mode SPE cartridges (see **Note #3**) with HPLC-grade methanol and HPLC-grade water at gravity.
14. Load the cartridges with the sample volume at 1drop s^−1^ approx.
15. Dry the cartridges passing air through it for 3 min.
16. Elute the cartridges with 4 mL of Mixture A (see **Note #4**). Then, circulate air for 2 min.
17. Elute the cartridges with 2 mL of Mixture B (see **Note #5**). Then, circulate air for 2 min.
18. Reduce the sample extracts (in glass tube) to the minimum volume with a gentle steam of N_2_.
19. Transfer the extracts from the glass tubes to HPLC vials.
20. Add 200 µL of methanol to the glass tubes and vortex for 15 s.
21. Transfer the methanol-extracts from glass tubes to the HPLC vials.
22. Repeat steps 19–20 three times.
23. Bring sample extracts (in HPLC vial) to dryness with a gentle N_2_ stream.
24. Reconstitute the residue with 0.5 mL of HPLC-grade methanol.
25. Add 0.5 mL of HPLC-grade water.

***Note #1***: In order to evaluate proper extraction of the method for each sample, add 20 µL of a solution 1 µg·mL-1 of selected compounds. Compounds added in the validation are shown in Table 1. For surrogate addition, a mixture of Clothianidine-d3, Caffeine-d3 and Benzotriazole-d4 in acetone was prepared.

**Table 1:** List of compounds selected for validation of the method. Linearity, matrix effect, recoveries and RSD at 2 different levels (20 and 150 ng g^−1^*d.w.*) are shown in whole body extract in krill and lantern fish matrices.

			KRILL					LANTERN FISH				
Compound	Class	LogP	Lin^1^	ME^2^	Rec. L ± RSD^3^	Rec. H ± RSD^4^	L.O.D.^5^	Lin^1^	ME^2^	Rec. L ± RSD^3^	Rec. H ± RSD^4^	L.O.D.^5^
Tyramine	Food additive	0.72	0.992	−4%	53±33	26±18	0.010	–	–	–	–	–
2-amino-Benzothiazole	Food additive	1.54	0.980	−69%	110±34	61±17	0.081	0.996	−27%	–	118±4	0.010
2-Benzothiazolesulfonic acid	Food additive	1.67	0.987	−86%	102±11	40±31	0.010	0.994	−39%	–	96	0.012
2‑hydroxy-Benzothiazole	Food additive	2.28	0.994	−60%	155	94	0.242	–	–	–	–	–
Dinoterb	Herbicide	3.42	0.968	−74%	50±10	52±12	0.034	0.927	−60%	55±7	43±41	0.010
Clothianidin	Insecticide	0.40	0.990	−61%	95±1	95±11	0.010	0.999	−63%	112±4	103±2	1.651
Thiamethoxam	Insecticide	−1.16	0.981	−47%	109±12	79±9	0.037	0.998	−50%	122±4	74±6	4.76
Tryptamine	Metabolite	1.38	0.977	−69%	45±21	40±7	0.122	0.997	−67%	54±2	27±4	0.580
Perfluorobutanesulfonate	Per-fluorinated	3.68	0.917	−27%	110±6	97±7	0.016	0.862	−28%	103±6	80±24	0.010
Perfluorobutanesulfinate	Per-fluorinated	7.03	1.000	−83%	115	97	0.010	0.994	−78%	100	98	0.010
Denatonium	Personal Care Prod.	0.09	0.918	−71%	123±51	120±42	3.68	0.965	−70%	68±35	52±46	0.260
Lauryl diethanolamide	Personal Care Prod.	3.94	0.992	−90%	90	93	0.010	0.999	−48%	–	105	0.148
Methyl paraben	Personal Care Prod.	1.87	0.979	−57%	-	-	0.078	0.977	−54%	139±4	113±21	0.030
N,N-Dimethyltetradecylamine	Personal Care Prod.	6.97	0.994	−66%	38	40	0.057	0.996	−60%	70	53	0.076
4 - Formylamino Antipyrine	Pharmaceutical	−0.41	0.982	–	–	–	1.18	0.991	–	76±24	238±65	3.65
Hydroxychloroquine	Pharmaceutical	3.77	0.941	38%	9 ± 46	10±66	0.078	0.991	10%	–	4 ± 37	15.79
Lopinavir	Pharmaceutical	6.26	0.991	−79%	83±14	95±1	0.063	0.998	−67%	115±4	108±2	12.84
Oseltamivir	Pharmaceutical	1.50	0.996	−68%	69±1	75±8	0.038	0.999	−66%	94±5	67±4	0.933
Pilocarpine	Pharmaceutical	−0.09	0.975	92%	78±3	68±1	10.67	0.996	82%	72±5	53±3	0.012
Ritonavir	Pharmaceutical	5.28	0.989	−57%	96±11	109±1	3.13	0.998	−52%	132±10	115±2	4.98
Salicylamide	Pharmaceutical	1.41	0.983	−53%	65±4	55±4	0.190	0.973	−50%	80±2	55±21	0.011
Bisphenol S	Plasticizer	1.83	0.994	−71%	106±3	99±2	0.014	0.982	−65%	110±8	90±12	0.719
Dimethyl phthalate	Plasticizer	1.64	0.854	−74%	83±7	62±12	0.016	0.914	−76%	80±2	41±10	1.01
Caffeine	Stimulant	−0.13	0.994	−60%	101±9	103±6	0.171	0.999	−56%	116±5	95±3	1.04
Nicotine	Stimulant	0.72	0.972	−39%	52±16	43±14	0.010	0.993	−43%	52±4	28±10	0.096
Tetradecylsulfate	Surfactant	6.46	0.995	−15%	87±70	–	0.574	–	–	–	–	–
Benzotriazole	UV-filter	1.34	0.994	−62%	107±5	105±9	0.019	1	−60%	127±5	99±2	0.156

1 Linearity.

2 Matrix effect in %.

3 Recoveries at 20 ng g^−1^and RSD given in %.

4 Recoveries at 150 ng/g and RSD given in %.

5 Limit of detection, given in ng g^−1^.

***Note #2:*** In order to prepare the solvents for the extractions, two individual solutions were prepared. For the first one (S1), weigh 19.213 g of citric acid and dissolve them in 1000 mL of HPLC water. For the second one (S2), weigh 14.705 g of tri-sodium citrate 2-hydrate and dissolve them in 1000 mL of HPLC water. Mix 118 mL of S1 with 82 mL of S2 and mix carefully. Then, add 200 mL volume of S1:S2 (59:41) mixture to 200 mL acetonitrile.

***Note #3****: The homemade* cartridges used were those previously described in the literature (Gago-Ferrero et al. [2]). Cartridges contains 0.2 g of *Sepra ZT*,0.1 g *Sepra ZTL-WCX*, 0.1 g *Sepra ZTL-WAX* and 0.15 g of *Isolute ENV+* from Biotage.

***Note #4*:** Mixture A has been prepared by mixing methanol, (470 mL), ethyl acetate (470 mL) and 32% ammonia solution (60 mL). Final concentration of ammonia is 2%.

**Note #5:** Mixture B has been prepared by mixing Methanol (490 mL), Ethyl Acetate (490 mL) and formic acid 88–90% (20 mL). Final concentration of formic acid is 1.8%.

## Instrumental analysis

After sample extraction, 10 µL of sample extracts are directly injected (avoiding filtration steps in order to minimize compound losses as much as possible) in the UPLC-HRMS instrument under the following chromatographic conditions:

**Table:** 

*LC parameters positive ionization mode*	
*Column*	*Acquity UPLC C18 column (100* *Å, 1.8* *µm, 2.1* *×* *100* *mm)*
*Column temperature*	*40* °*C*
*Mobile phase A*	*0.1% formic acid in methanol*
*Mobile phase B*	*0.1% formic acid in water*
*Sample volume*	*10 µL*
*Gradient*	*(%A): Initial 5%, 75% at 7* min*, 100% at 10* min*, 100% at 15* min*, 5% at 17* min *and 5% at 23* min*.*

**Table:** 

*LC parameters negative ionization mode*	
*Column*	*Acquity UPLC C18 column (100* *Å, 1.8* *µm, 2.1* *×* *100* *mm)*
*Column temperature*	*40* °*C*
*Mobile phase A*	*5* *mM ammonium acetate in methanol*
*Mobile phase B*	*5* *mM ammonium acetate in water*
*Sample volume*	*10 µL*
*Gradient*	*(%A): Initial 5%, 50% at 3* min*, 90% at 6* min*, 100% at 13* min*, 100% at 17* min*, 5% at 18* min*, and 5% at 20* min*.*

For HRMS, parameters applied for the analysis were:

**Table:** 

*HRMS parameters for positive (+) and negative (-) ionization modes*	
*Spray voltage*	*3000 (+), 2800 (-)*
*Capillary temperature*	*350* °*C (+) & (-)*
*Sheath gas*	*40 (+) & (-)*
*Aux Gas*	*10 (+) & (-)*
*Max. Spray current*	*100 (+) & (-)*
*Probe heater temp.*	*350 (+) & (-)*
*S-Lens RF Level*	*60 (+) & (-)*

Samples were analysed in a Q-Exactive Orbitrap mass analyser (Thermo Scientific) in Data Dependent acquisition (DDA), acquiring MS/MS for the 5 most intense ions and Data Independent (DIA) analysis at 35 eV of collision energy in both cases for MS/MS and high energy functions.

### Quality assurance and quality control

In order to prevent background contamination, all glassware was previously washed with ethanol and acetone and heated overnight at 450 °C. Furthermore, nitrile gloves were worn during the process. For avoiding compounds photodegradation during all the process, solutions were in amber bottles and stored in freezer at −20 °C in the dark.

Additionally, procedural blank samples were processed following all the steps of the extraction protocol (except step 3). These blanks were used for ensuring there has not been any background contamination during the sample treatment procedure.

A mixture of selected compounds (Table 1) prepared in acetone was added directly into the extraction in step 4, allowing solvent evaporation keeping the tube at room temperature for 30–60 min.

## Method validation

The method performance was evaluated with a set of compounds including pharmaceuticals, personal care products, herbicides, food additives and other industrial chemicals. The selected compounds comprised a wide range of polarity (log*P* comprised between −1.16 and 7.03) to ensure a good method performance in both the extraction and the clean-up steps in each sample (see Table 1).

The linear dynamic range, based on linear regression calibration curves prepared in each matrix, was studied in standard solution at four different concentration levels, ranging from 25 to 500 µg L^−1^. All areas were integrated using the extracted ion chromatogram for the corresponding *m/z* of the parent ion with a window of 5 ppm. A good linearity range was obtained for almost all compounds. Out of the evaluated 27 compounds, 18 and 20 showed coefficient R^2^ > 0.98 in krill and lantern fish, respectively.

In order to evaluate the extraction efficiency absolute recoveries were determined by spiking both krill and lantern fish with a standard mixture at two concentrations (20 and 150 ng g^−1^). Recovery values between 50 and 130% were obtained for >70% of compounds. Few compounds showed values out of this range indicating poor extraction and/or important matrix effects. Overall results were very satisfactory considering the wide range of physicochemical properties covered by the selected compounds and the complexity of the biologic matrices of interest. The method precision was estimated with repeatability values, in terms of %RSD, for three experimental replicates, showing values below 20% in most cases.

Limits of detection were estimated based on the signal to noise (S/N) ratios of low concentration matrix calibration standards. The method limits of detection (equivalent of S/*N* = 3) obtained varied from 0.01 to 10.67 ng g^−1^ *d.w.* and from 0.01 to 15.79 ng g^−1^ *d.w.* for krill and lantern fish, respectively. Results show the overall good sensitivity of the methodology for the screening of a wide range of xenobiotics in complex biota samples.

Matrix effects (signal suppression or enhancement) were also calculated for the selected compounds in both matrices according to Eq. (1):

(1) $$Matrix\;effect\;\left(\%\right):\left(\frac{\left(Area\;matrix-Area\;blank\right)}{Area\;solvent}-1\right)x100$$

Where *Area matrix* corresponds to the response given by the instrument for the selected compound in a spiked matrix sample, *Area blank* is the response given by the instrument in a non-spiked matrix samples and *Area solvent* is the response given by the instrument in a solvent spiked sample.

Almost all compounds showed ion suppression, with values ranging from −4% to −92% in krill and from −14% to −82% in lantern fish extracts. Only hydroxychloroquine and pilocarpine showed ion enhancement in both matrices.

Overall, the developed methodology showed a very good performance for the determination of a wide range of xenobiotics in biological matrices. This is particularly certain considering the differences in polarity and other physicochemical properties of the selected compounds for the validation step. However, if the final aim of the user is to provide reliable quantitative data of new compounds identified through suspect and non-target approaches, it is advisable to fully validate the methodology for the newly identified substances.
